# Complete plastid genome sequence of *Primula sinensis* (Primulaceae): structure comparison, sequence variation and evidence for *accD* transfer to nucleus

**DOI:** 10.7717/peerj.2101

**Published:** 2016-06-28

**Authors:** Tong-Jian Liu, Cai-Yun Zhang, Hai-Fei Yan, Lu Zhang, Xue-Jun Ge, Gang Hao

**Affiliations:** 1 College of Life Sciences, South China Agricultural University, Guangzhou, China; 2 Key Laboratory of Plant Resources Conservation and Sustainable Utilization, South China Botanical Garden, Chinese Academy of Sciences, Guangzhou, China

**Keywords:** Primula sinensis, Chloroplast genome, Illumina sequencing, Gene transfer

## Abstract

Species-rich genus *Primula* L. is a typical plant group with which to understand genetic variance between species in different levels of relationships. Chloroplast genome sequences are used to be the information resource for quantifying this difference and reconstructing evolutionary history. In this study, we reported the complete chloroplast genome sequence of *Primula sinensis* and compared it with other related species. This genome of chloroplast showed a typical circular quadripartite structure with 150,859 bp in sequence length consisting of 37.2% GC base. Two inverted repeated regions (25,535 bp) were separated by a large single-copy region (82,064 bp) and a small single-copy region (17,725 bp). The genome consists of 112 genes, including 78 protein-coding genes, 30 tRNA genes and four rRNA genes. Among them, seven coding genes, seven tRNA genes and four rRNA genes have two copies due to their locations in the IR regions. The *accD* and *infA* genes lacking intact open reading frames (ORF) were identified as pseudogenes. SSR and sequence variation analyses were also performed on the plastome of *Primula sinensis*, comparing with another available plastome of *P. poissonii*. The four most variable regions, *rpl36–rps8*, *rps16–trnQ*, *trnH–psbA* and *ndhC–trnV*, were identified. Phylogenetic relationship estimates using three sub-datasets extracted from a matrix of 57 protein-coding gene sequences showed the identical result that was consistent with previous studies. A transcript found from *P. sinensis* transcriptome showed a high similarity to plastid *accD* functional region and was identified as a putative plastid transit peptide at the N-terminal region. The result strongly suggested that plastid *accD* has been functionally transferred to the nucleus in *P. sinensis*.

## Introduction

Chloroplast is one of the most important organelles in green plant cells, and plays a central role in plant photosynthesis. Sequence data from chloroplast genomes (or plastomes) has been widely used in phylogenetic studies, because of its recombination-free and maternal inheritance (Graham & Olmstead, 2000). More importantly, they are structurally highly conserved, which facilitates PCR primer design and sequencing (Shaw et al., 2005; Shaw et al., 2014). However, obtaining accurate phylogenies using a small standardized set of chloroplast genes is challenging in some rapidly evolving plant groups, such as *Gaertnera* (Malcomber, 2002). Recently, chloroplast phylogenomics has been successfully used in several plant groups since the advent of 454 and/or Illumina technologies (Moore et al., 2006; Cronn et al., 2008; Ma et al., 2014; Ruhfel et al., 2014; Parks, Cronn & Liston, 2009), which offer an increasingly easy-to-access source of characters to resolve ambiguous phylogenetic relationships in some rapidly evolved plant groups (Folk, Mandel & Freudenstein, 2015; Wysocki et al., 2015).

The structure or functional change for the chloroplast genome is interesting as well. The chloroplast is considered to be a descendant of cyanobacterium-like progenitors (Raven & Allen, 2003). Since its endosymbiotic origin, the size of the chloroplast genome has been greatly reduced (Timmis et al., 2004). This shrunken genome is the consequence of the loss or transfer of genes to the nucleus (Martin et al., 2002). Loss of genes has been found in many lineages of angiosperms (Blazier, Guisinger & Jansen, 2011; Li et al., 2013). Meanwhile, only four genes transferred to nucleus have been confirmed by several studies, including *infA*, *accD*, *rpl22* and *rpl32* (Millen et al., 2001; Ueda et al., 2007; Magee et al., 2010; Jansen et al., 2011; Park, Jansen & Park, 2015). To better understand this transfer, it is necessary to explore more data from both the plastid and nucleus at a wide range of angiosperms.

The genus *Primula* L. is one of the largest genera in the family Primulaceae, and it was characterized by a rapid speciation at East Himalaya-Hengduan Mts. Region (Hu, 1994; Richards, 2003; Yan et al., 2015). Understanding the chloroplast genome of this genus will benefit us in constructing a solid phylogeny of the genus in the future. However, the complete chloroplast sequences of species in this genus still have been poorly understood except for a recently released chloroplast genome of *P. poissonii* Franch. without additional analyses (Yang, Li & Li, 2014).

In this study, we released a complete chloroplast genome of an endemic *Primula* species in China, *P, sinensis* Sabine ex Lindley, by using high-throughput sequencing technology. To start with this plastome sequence, firstly, we characterized gene content, sequence variation, and compared with other related species, which will facilitate further phylogenetic studies of the genus. Secondly, we verified the *accD* gene lacking intact open reading frames (ORF) from *P. sinensis* plastid and search clue in its transcriptome to get lines of evidences for functional transfer to nucleus.

## Materials and Methods

### Library preparation and Illumina sequencing

Fresh leaves of *P. sinensis* were collected from the South China Botanical Garden, Chinese Academy of Sciences (CAS). Modified CTAB method was used to isolate whole-genome DNA (Porebski, Bailey & Baum, 1997). RNAs in the initial extracts was digested by RNase A to acquire pure genomic DNA. Eight primer pairs involved in this study were designed according to Yang, Li & Li (2014), to amplify the whole chloroplast genome sequence. Primers were designed to cover inverted repeat region only once. A region of approximately 16 kb was amplified by each primer pair. Long-range PCR was performed with 25 μl reaction system by using Primestar GXL DNA polymerase (TaKaRa Bio., Dalian, China). Reactions were initially denatured for 1 min at 95 °C, followed by 35 cycles of 10 sec at 94 °C, 1 min at 60 °C, and 15 min at 68 °C. Eventually the additional extension step performed on 5 min at 68 °C.

PCR products were mixed to build pair-end library using Nextera XT DNA Library Prep Kit (Illumina Inc., San Diego, CA, USA). PCR products mixture was fragmented into ∼300 bp size by the Nextera XT transposome. Library Sequencing acquired 2× 250 bp paired reads using Illumina Miseq Desktop Sequencer at Kunming Institute of Botany, CAS. All reads data were deposited to NCBI SRA database with an accession number SRP068226.

### Plastome assembly and annotation

Reads were assembled using CLC Genomics Workbench v7.5.1 (CLC Bio., Aarhus, Denmark) after removing adaptors and trimmed low quality reads. Assembly was conducted twice separately with two different k-mer value 60 and 64. Contigs generated by assembling were subjected to BLAST searches against the complete chloroplast sequence of *P. poissonii* (NC_024543). Then, relative position and direction of each hitting contig were determined. Subsequently, hitting contigs were assembled manually to acquire complete chloroplast sequence in Geneious R7 (Biomatters, Auckland, New Zealand). The resulting plastome sequence was used as the reference, which was subsequently verified by remapping initial reads. Regions of four SC-IR junctions were identified by Sanger sequencing using four pair primers. Primer sequences used in this study can be found (Table S1).

Plastome annotation was performed using DOGMA (http://dogma.ccbb.utexas.edu/) (Wyman, Jansen & Boore, 2004) and compared with other Primulaceae species in alignment matrix. Annotations of each gene was adjusted to appropriate start and stop codons in accordance with the genetic codon for plant plastid. Incomplete genes identified from *P. sinensis* were verified by Sanger sequencing. The names of a few genes were updated according to the latest study, including *ycf3* to *pafI* and *ycf4* to *pafII* (Wicke et al., 2011). The annotated chloroplast genome sequence was submitted to GenBank (accession number: KU321892). Finally, a circular genome map was illustrated using OGDRAW (http://ogdraw.mpimp-golm.mpg.de/) (Lohse et al., 2013).

### Sequence variance and SSR analysis

To further identify highly variable regions in chloroplast genome of *Primula*, sequences of *P. sinensis* and *P. poissonii* were compared according to variability (Pi). Alignment was performed with MAFFT version 7 (Katoh & Standley, 2013). The genetic diversity (Pi) were calculated in each split regions (400 bp) of alignment using DnaSP version 5 (Librado & Rozas, 2009). The adjacent split regions were overlapped each other with 300 bp. Gaps in the alignment were excluded from analysis. With the variance of recommended barcode *rbcL* + *matK* + *ITS* as reference (Yan et al., 2015), the top 40 regions with most variable sites, whose aligned lengths are longer than 200 bp were extracted for the next informative character (PICs) analysis. We followed the method of Shaw et al. (2005) to count manually the numbers of nucleotide substitutions and indels for each regions and plot them in Fig. 3.

To detect and locate simple sequence repeats (SSRs), GMATo v1.2 (Wang, Lu & Luo, 2013) was used to screen the chloroplast sequence of two *Primula* species. The parameter settings of mononucleotide and dinucleotide to hexanucleotide were at least eight repeat units and four repeat units, respectively.

### RNAseq and *accD* gene characterizing

Total RNA was extracted using a modified CTAB method. Quantified total RNA (concentration ≥ 100 ng/μL; rRNA ratio ≥ 1.5) was delivered to Majorbio (Shanghai, China), where cDNA sequencing was performed with Illumina Hiseq4000 platform. Raw data were filtered and deposited in the Sequence Reads Archive (SRA) database under accession number SRX1665905. The cleaned reads were assembled de novo using Trinity with the default parameters to obtain 48,887 unisequences. Eight different software, namely TargetP (Emanuelsson et al., 2000), Protein Prowler (Hawkins & Bodén, 2006), BacelLo (Pierleoni et al., 2006), CELLO2GO (Yu et al., 2014), Euk-mPLoc2 (Chou & Shen, 2010), EuLoc (Chang et al., 2013), HybridGO-Loc (Wan, Mak & Kung, 2014), and Predotar (Small et al., 2004), were used to identify the subcellular location signals from N-terminal sequence of putative nuclear-encoded protein.

### Phylogenetic analysis

Plastome of *P. sinensis* together with eight other plastomes published previously from different genera in Ericales was involved in this phylogenetic analysis, and *Agrostemma githago* from Caryophyllales was used as outgroup. All plastomes used in this study are available in GenBank (Table S2). A total of 57 plastid protein-coding genes were concatenated to generate three data sets according to the different strategies, such as all CDS, codon1+2 and condon3, respectively. On the other hand, the fourth datasets were generated including 30 pt-*accD* genes from six families in Ericales and putative n-*accD* gene in *P. sinensis*. All sequences from four datasets were aligned using the default option implemented in MAFFT version 7 (Katoh & Standley, 2013). Maximum likelihood (ML) trees was constructed with RAxML (RAxML-VI-HPC, http://www.trex.uqam.ca/) using GTR + Γ nucleotide substitution model (Stamatakis, 2006). Branch supports were assessed with 500 bp replicates.

## Results and Discussion

### Chloroplast genome assembly

The *P. sinensis* chloroplast genome was sequenced using Illumina Miseq sequencer, producing total number of 2× 250 bp pair-end reads 2,640,572. The mean coverage depth is about 3838.8×, ranging from 40× minimum to 25814× maximum. Two assemblies with different k-mer values successfully generated identically complete sequence with no gaps. Six contigs from assembly with k-mer value 60 were matched to reference plastome sequence, which was used to determine relative position and direction respectively. A new draft chloroplast genome was generated by identifying overlap regions manually. The draft genome was then checked and corrected according to quality and coverage of each base position by reads remapping (Fig. S1). The annotated genome was deposited into GenBank under the accession number KU321892.

### Features of the *P. sinensis* chloroplast genome

The chloroplast genome of *P. sinensis* has a total length of 150,859 bp. It is divided into three parts: 82,064 bp of large single-copy (LSC) region, 17,725 bp of small single-copy (SSC) region, and two inverted repeat (IR) regions with 25,535 bp of one copy in length. The nucleotide composition of this genome has a GC content of 37.2%. Comparative analysis revealed that the genome structure of *P. sinensis* shared a high similarity structure to other Primulaceae species (*P. poissonii* NC_024543, *Lysimachia coreana* NC_026197, *Ardisia polysticta* KC465962) (Ku, Hu & Kuo, 2013; Son & Park, 2014) (Table 1).

**Table 1:** Comparison of the general features of four plastomes in Primulaceae.

	*Primula sinensis* (KU321892)	*Primula poissonii* (NC_024543)	*Lysimachia coreana* (NC_026197)	*Ardisia polystica* (KC465962)
**Plastome size**				
Total size	150,859	151,664	155,386	156,506
LSC size	82,064	83,444	85,229	86,078
SSC size	17,725	17,822	17,951	18,328
IR size	25,535	25,199	26,103	26,050
**Base content (%)**				
Total A content	31.1	31.1	31.1	31.2
Total T content	31.7	31.8	31.8	31.8
Total C content	19	18.8	18.9	18.9
Total G content	18.2	18.2	18.2	18.2
LSC GC content	35.2	34.9	35	34.9
SSC GC content	30.5	30.1	30.5	30.2
IR GC content	42.8	42.9	42.9	43
Total GC content	37.2	37	37.1	37.1
**Number of genes**				
Total	112	113	113	114
Protein encoding	78	79	79	80
tRNA	30	30	30	30
rRNA	4	4	4	4

**Figure 1:**
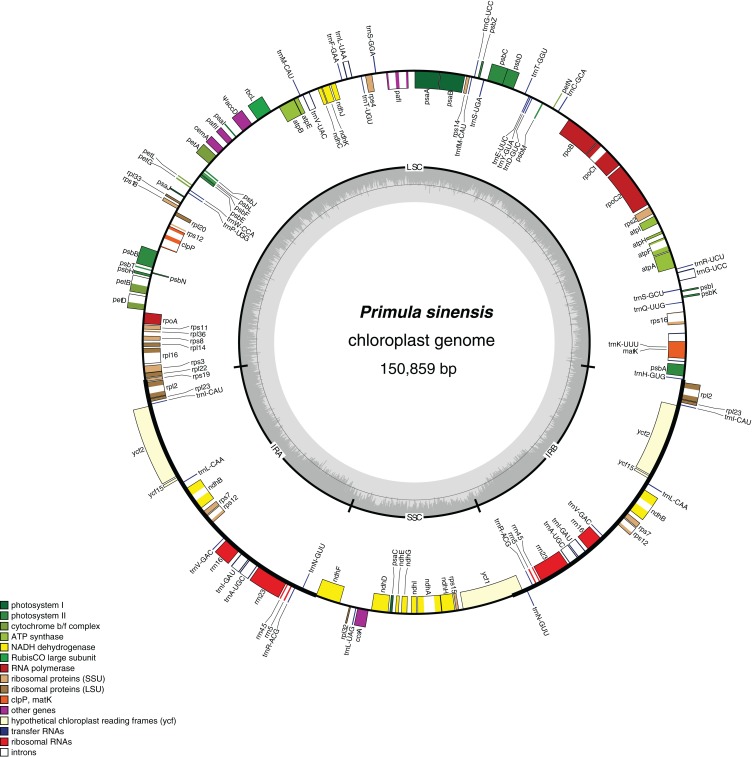
Chloroplast genome map of *Primula sinensis* (GenBank accession number KU321892). Genes on the outside of the outer circle indicate the clockwise direction of transcription; those on the inside indicate the counterclockwise direction. The bar graphs on the inner circle reveal GC content in dark grey with the 50% threshold line.

A total of 112 genes were detected in this chloroplast genome, which could be classified into 78 protein-coding genes, 30 tRNA genes and four rRNA genes. Among them, seven coding genes, seven tRNA genes and four rRNA genes have two copies due to their locations in the IR regions. As we expected, split genes also exist in this plastome. Among them, 15 genes contain an intron and two genes have two introns (Table 2; Fig. 1).

**Table 2:** Gene contents in *Primula sinensis* chloroplast genome (112 genes, two pseudogenes).

Category	Class	Gene
**Genetic apparatus**	DNA-dependent RNA polymerase	rpoA, rpoB, rpoC1*, rpoC2
	Maturase	*matK*
	Large ribosomal subunits	rpl2*(x2), rpl14, rpl16*, rpl20, rpl22, rpl23(x2), rpl32, rpl33, rpl36
	Small ribosomal subunits	*rps2, rps3, rps4, rps7(x2), rps8, rps11,* ***rps12***, rps14, rps15, rps16*, rps18, rps19
	Protease	clpP**
	Ribosomal RNAs	*rrn4.5(x2), rrn5(x2), rrn16(x2), rrn23(x2)*
	Transfer RNAs	trnH-GUG, trnK-UUU*, trnQ-UUG, trnS-GCU, trnG-UCC*, trnR-UCU, trnC-GCA, trnD-GUC, trnY-GUA, trnE-UUC, trnT-GGU, trnS-UGA, trnG-GCC, trnfM-CAU, trnS-GGA, trnT-UGU, trnL-UAA*, trnF-GAA, trnV-UAC*, trnM-CAU, trnW-CCA, trnP-UGG, trnI-CAU(x2), trnL-CAA(x2), trnV-GAC(x2), trnI-GAU*(x2), trnA-UGC*(x2), trnR-ACG(x2), trnN-GUU(x2), trnL-UAG
**Light-dependent photosynthesis**	Photosystem I	*psaA, psaB, psaC, psaI, psaJ*
	Photosystem II	*psbA, psbB, psbC, psbD, psbE, psbF, psbH, psbI, psbJ, psbK, psbL, psbM, psbN, psbT, psbZ*
	NAD(P)H dehydrogenase complex	ndhA*, ndhB*(x2), ndhC, ndhD, ndhE, ndhF, ndhG, ndhH, ndhI, ndhJ, ndhK
	F-type ATP synthase	atpA, atpB, atpE, atpF*, atpH, atpI
	PS I assembly factor	pafI**, pafII
	Cytochrome b6/f complex	petA, petB*, petD*, petG, petL, petN
**Light-independent photosynthesis**	Inner membrane protein	*cemA*
	Cytochrome c biogenesis protein	*ccsA*
	Large subunit of Rubisco	*rbcL*
**Other**	Function unknown	*ycf1, ycf2(x2), ycf15(x2)*
**Pseudogene**	Subunit of acetyl-CoA-carboxylase	*accD*
	Translation initiation factor	*infA*

**Notes:**

* Represent gene with one intron.

** Represent gene with two introns; “x2” represent gene location within IR region; Bold represent genes with alternative splicing.

**Figure 2:**
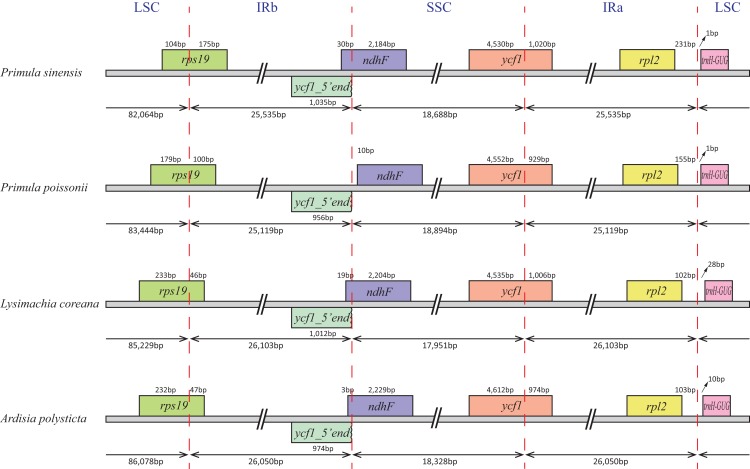
Comparison of the SC-IR boundary of four plastomes from Primulaceae.

In particular, the *rps12* gene is interrupted into three pieces which resulted in trans-splicing because of its first exon located in LSC, while its second and third exons located in IRs (Hildebrand et al., 1988). Notably, the phenomena that two coding regions sharing overlapped sequence with different reading frames was found in *psbD* and *psbC*, *atpE* and *atpB*, and *rps3* and *rpl22*. In addition, three genes, *rps19*, *ndhF* and *ycf1,* cross the LSC-IRb, IRb-SSC, SSC-IRa boundary, respectively. Furthermore, the *ndhF* 3′-terminal sequence shares the region in the IRb with the rest of ycf1 5′-terminal sequence, while the IRb-SSC boundary of *P. poissonii* was separated from the start codon of *ndhF* with 10 bp length (Fig. 2). Significantly, the *accD* in *P. sinensis* were identified as pseudogene with an extremely reduced ORF. Meanwhile, the *infA* lacked intact ORF in *P. sinensis, P. poissonii* and *L. coreana*, which strongly indicated that pseudogenization remains occurred in certain angiosperm groups. Further studies are necessary to focus on the mechanism of occurrence of these pseudogenes and applications of orthologous genes for phylogenetic analysis.

### SSR analysis of *Primula sinensis*

Perfect SSRs were screened in *P. sinensis* and *P. poissonii* conducted by GMATo v1.2. Mono-, di- and tri-nucleotide repeats were found in both species (Table 3). The total number of SSRs in *P. sinensis* chloroplast genome is 193, of which 148 homopolymers, 44 dipolymers and 1 tripolymers are respectively found in this genome. Among them, 85.49% SSRs are only composed of A/T bases. Similar quantity level and base proportion of SSRs were also found in *P. poissonii* (Tables S3–S5).

**Table 3:** Number of chloroplast SSRs present in two *Primula* species.

Taxon	Length (bp)	GC%	Number of SSRs			
			Homo (> 8 units)	Di (> 4 units)	Tri (> 4 units)	Total
*Primula sinensis*	150,859	37.20%	148	44	1	193
*Primula poissonii*	151,664	37%	129	46	2	177

### Sequence variation in two *Primula* plastomes

Although the chloroplast genome of *P. sinensis* has a similar genome structure in gene contents and orders in comparison with *P. poissonii*, there are considerable differences in noncoding regions, especially in intergenic sequence (IGS) regions. Highly divergence regions are potential molecular genetic markers for population genetics studies. We therefore compared the regional divergence of chloroplast genome sequences of these two *Primula* species. The Pi value generated by DnaSP version 5 was used to indicate the level of divergence between *P. sinensis* and *P. poissonii* (Fig. S2). Then, the top 40 regions with most variable sites, with aligned length longer than 200 bp, were extracted from the alignment for further analysis (PIC calculation).

**Figure 3:**
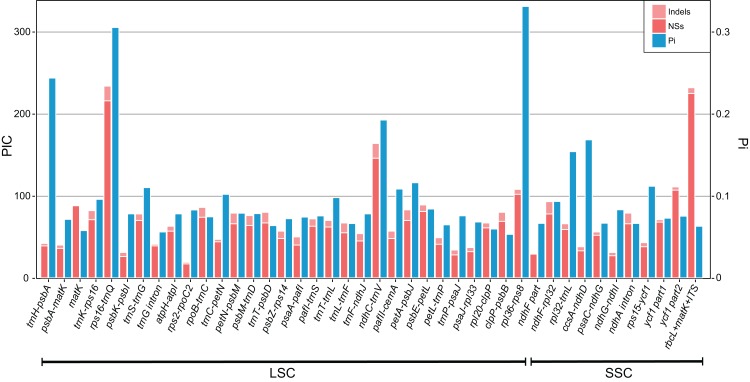
Bar plot that compares potential marker regions with PICs and genetic diversity (Pi). PICs were determined as the sum of nucleotide substitutions and indels. Barcode *rbcL* + *matK* + *ITS* was used here as a reference as proposed previously for the barcoding analysis of genus *Primula* (Yan et al., 2015).

The result shows the genetic diversity (Pi) varied from 0–0.47 (Table S6). Most of the variation occurs in the non-coding regions of the LSC and SSC, while less variable characters were found in IRs. The four most variable loci, namely *rpl36–rps8*, *rps16–trnQ*, *trnH*–*psbA* and *ndhC–trnV*, were identified. All of these loci have been reported previously (Shaw et al., 2007). Remarkably, *rpl36*–*rps8* exhibits the improved degree of variation. A total of 41 regions had been extracted to calculate their PICs (Fig. 3). Comparing the result with sequence variance, PICs are considered to be affected by sequence length apparently. For example, the *trnH-psbA* spacer has the lower number of PICs as its aligned length is only 213 bp. In contrast, *ycf1* have the high PICs with the sequence length coming up to 2,500 bp in total. However, difficulties in primer designing and PCR amplification limit them (such as *ycf1*) for further phylogenetic use. We therefore recommend *rpl32–trnL*, *trnS–trnG*, *pafII–cemA*, *trnC–petN*, *trnT–trnL*, *trnK–rps16* as efficient chloroplast markers, considering their balanced PICs and length size, although additional researches of their utilities are necessary in future.

### Pseudogenization of pt-*accD* and evidence for transfer to nucleus

*AccD* gene encodes β subunit of the Acetyl-CoA Carboxylase, which is unique related to fatty acid synthesis within the chloroplast. Gene knockout experiments in tobacco indicated that *accD* gene function is indispensable, suggesting it should be an essential gene (Kode et al., 2005). So far, several researches have reported that lack of a plastid *accD* (pt-*accD*) gene or pseudogenization of pt-*accD* are widely present in multiple distant lineages, including Acoraceae, Campanulaceae, Ericaceae, Fabaceae, Geraniaceae and Poaceae (Goremykin et al., 2005; Guisinger et al., 2008; Haberle et al., 2008; Magee et al., 2010; Fajardo et al., 2013; Harris et al., 2013; Martínez-Alberola et al., 2013), which implied the lack of pt-*accD* gene or events of pseudogenization occur independently. Owing to its fundamental function in plastid development, there should be some equivalent genes replaced functionally in other subcellular structures. In recent years, a few case of nuclear encoded *accD* genes (n-*accD*) originated from plastid have been found in different taxa, such as *Sciadopitys verticillata* (Sciadopityaceae) (Li et al., 2016), *Trifolium repens* (Fabaceae) (Magee et al., 2010), *Trachelium caeruleum* (Campanulaceae) (Rousseau-Gueutin et al., 2013). These n-*accD* genes share a similar 3′-terminal region with pt-*accD* genes which corresponds to the carboxylase domain, while the 5′-terminal regions are completely different from pt-*accD*. Products of these n-*accD* have a putative transit peptide at the 5′-terminus. The functional prediction suggests that the transit peptide guides the protein product relocation to chloroplast. Fluorescence microscopy showed that n-ACCD-GFP fusion protein was imported in plastids contained in tobacco guard cells (Rousseau-Gueutin et al., 2013), which provided strong evidence that nuclear encoded *accD* gene still returns to plastid to play its roles as the same as other subunits of nuclear-encoded plastid ACCase do.

In this study, plastid *accD* locus in *Primula sinensis* shows the truncated gene with incomplete ORF. We found a portion of pt-*accD* gene near the start position was absent with about 400 bp in length by comparing with other plastomes from Primulaceae. This deletion has been verified by PCR amplification using a pair of primers located on both flanks. Reads mapping also showed high coverage levels on flanking regions of the deletion. Due to the presence of the deletion, pt-*accD* coding sequence was terminated prematurely with the introduction of the stop codon. The remaining ORF with residual sequences does not include the conserved functional region. Actually, pt-*accD* has become a pseudogene, with a deletion involved (Figs. S3 and S4).

**Figure 4:**
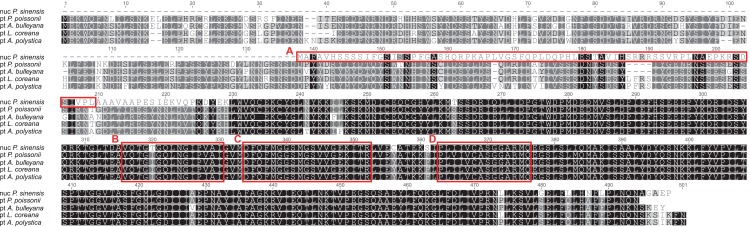
Alignment of the putative nuclear *accD* from *Primula sinensis* and the plastid *accD* from *Primula poissonii*, *Androsace bulleyana*, *Lysimachia coreana* and *Ardisia polystica.* (A) indicates the putative transit peptide at the N-terminal region of nuclear *accD*. (B, C, D) indicates three putative motifs, acetyl-CoA binding sites, carboxybiotin binding sites and carboxyltransferase catalytic sites, respectively.

Owing to its important function in plastid development, *accD* should probably be transferred into other subcellular structures, and still retained its catalytic activity (Rousseau-Gueutin et al., 2013). We therefore checked the transcriptome of *P. sinensis* using *P. poissonii* pt-*accD* as reference. The result of blast searching showed only one transcript, which was highly identical to the plastid *accD* functional region of *P. poissonii*. This transcript contained an intact ORF encoding a protein with 362 amino acids. In comparison with pt-*accD* sequences of other related species (such as *Androsace bulleyana*, *Lysimachia coreana* and *Ardisia polystica*, Fig. 4), the ORF of this transcript is different from pt-*accD* coding regions of other species. However, their C-terminal regions are conserved, which all contained three putative motifs, such as acetyl-CoA binding sites, carboxybiotin binding sites and carboxyltransferase catalytic sites (Lee et al., 2004). In contrast, N-terminal of the ORF shows no similarity against any plastid-encoded sequences (Fig. 4). In order to test whether the software (TargetP and Protein Prowler) affect the results of subcellular localization prediction of this putative protein-encoded sequence, we also predicted the protein sorting signals using other six software (BacelLo, CELLO2GO, Euk-mPLoc2, EuLoc, HybridGO-Loc, and Predotar). Our conclusion of the protein subcellular localization was confirmed by identical results provided by other software (data not shown). Prediction results showed a chloroplast transit peptide of 72 residues length located at the N-terminus (Fig. 4). The chloroplast transit peptides of nuclear-encoded plastid proteins (NUPTs) are necessary for targeting and import of proteins into chloroplasts. It is strongly suggested that plastid *accD* has been functionally transferred to the nucleus in *P. sinensis*. This is the fourth report in angiosperms (to our knowledge) for the transferability of *accD* gene with lines of solid evidences.

**Figure 5:**
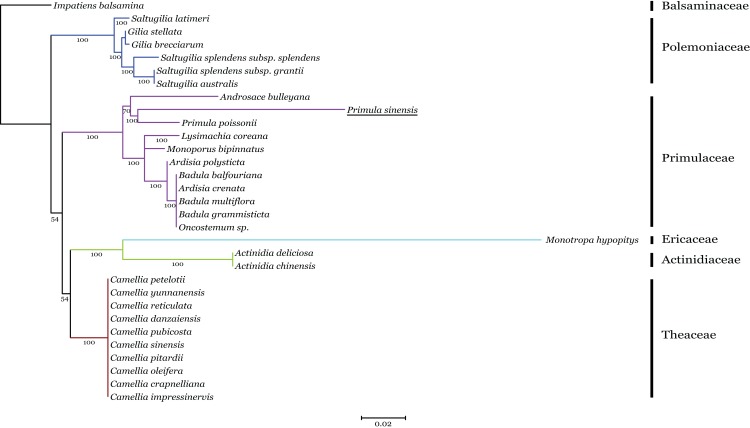
Phylogenetic analysis of *accD* sequences from six families in Ericales using the Maximum likelihood. The underline indicates the putative n-*accD* gene of *Primula sinensis*.

Phylogenetic analysis of the n-*accD* transcript in *P. sinensis* with pt-*accD* from other 30 species, which belongs to six families in Ericales, was performed by RAxML. This dataset contained multiple alignment of 31 sequences of the C-terminal functional regions, since the regions are relatively conserved as discussed above. The maximum likelihood tree showed n-*accD* from *P. sinensis* was located within the clade of the Primulaceae with *P. poissonii* as the sister group (Fig. 5). Considering the close relationship between *P. sinensis* and *P. poissonii*, this result probably indicated that *accD* of *P. sinensis* might have transferred to nucleus recently. Available data showed that complete pt-*accD* genes have been lost functionally from three species, *Arbutus unedo* (JQ067650), *Vaccinium macrocarpon* (JQ757046), and *Chamaedaphne calyculata* (KJ463365) in Ericaceae. It is likely that the missing or pseudogenization of pt-*accD* genes occurred accidently and independently in Ericales.

### Phylogenetic analysis based on plastome sequence

**Figure 6:**
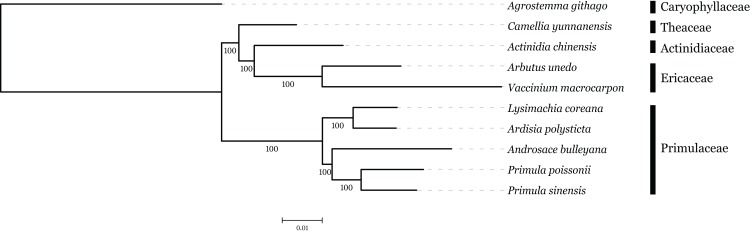
The ML tree produced by chloroplast genome sequence of *Primula sinensis* and other nine species. Numbers associated with each node are bootstrap values with 500 replicates.

The family interrelationships within the large order Ericales, constrained by the data available, have remained unclear (Anderberg, Rydin & Källersjö, 2002; Bremer et al., 2002; Geuten et al., 2004). Recently, advances in high-throughput sequencing have provided a large amount of data, which was an improvement of the phylogenetic resolution (Wen et al., 2013; Yang et al., 2015). With the expectation, nine plastome sequences represented different genera in Ericales involved in phylogenetic analysis, with *Agrostemma githago* as outgroup. Phylogenetic relationships were inferred using 57 plastid protein-coding genes. As we know, the third base position of codon evolves faster than the rest of two positons with higher substitution rate. ML trees were produced by using three different datasets, all coding sequence, codon 1 + 2 and codon3, respectively. The result showed that topologies of three datasets were highly congruent with one another and all nodes were well supported (Fig. 6). Primuloideae and Myrsinoideae within Primulaceae s. l. fall in two branches separately with strong supports. Primulaceae s. l. was placed as sister to the clade that comprising Theaceae, Actinidiaceae, and Ericaceae. The phylogenetic positions of these groups are in agreement with recent studies (Stevens, 2012; Zhang et al., 2015).

## Supplemental Information

10.7717/peerj.2101/supp-1 Supplemental Information 1 Supplementary Materials.

## Additional Information and Declarations

### DNA Deposition

The following information was supplied regarding the deposition of DNA sequences: GenBank accession number: KU321892.

### Data Deposition

The following information was supplied regarding data availability: NCBI SRA database: SRP068226 and SRX1665905.

## References

[ref-1] Anderberg AA, Rydin C, Källersjö M (2002). Phylogenetic relationships in the order Ericales s.l.: analyses of molecular data from five genes from the plastid and mitochondrial genomes. American Journal of Botany.

[ref-2] Bremer B, Bremer K, Heidari N, Erixon P, Olmstead RG, Anderberg AA, Källersjö M, Barkhordarian E (2002). Phylogenetics of asterids based on 3 coding and 3 non-coding chloroplast DNA markers and the utility of non-coding DNA at higher taxonomic levels. Molecular Phylogenetics and Evolution.

[ref-3] Blazier J, Guisinger MM, Jansen RK (2011). Recent loss of plastid-encoded ndh genes within *Erodium* (Geraniaceae). Plant Molecular Biology.

[ref-4] Chang TH, Wu LC, Lee TY, Chen SP, Huang HD, Horng JT (2013). EuLoc: a web-server for accurately predict protein subcellular localization in eukaryotes by incorporating various features of sequence segments into the general form of Chou’s PseAAC. Journal of Computer-Aided Molecular Design.

[ref-5] Chou K-C, Shen H-B (2010). A new method for predicting the subcellular localization of eukaryotic proteins with both single and multiple sites: Euk-mPLoc 2.0. PLoS ONE.

[ref-6] Cronn R, Liston A, Parks M, Gernandt DS, Shen R, Mockler T (2008). Multiplex sequencing of plant chloroplast genomes using Solexa sequencing-by-synthesis technology. Nucleic Acids Research.

[ref-7] Emanuelsson O, Nielsen H, Brunak S, von Heijne G (2000). Predicting subcellular localization of proteins based on their N-terminal amino acid sequence. Journal of Molecular Biology.

[ref-8] Fajardo D, Senalik D, Ames M, Zhu H, Steffan SA, Harbut R, Polashock J, Vorsa N, Gillespie E, Kron K, Zalapa JE (2013). Complete plastid genome sequence of *Vaccinium macrocarpon*: structure, gene content, and rearrangements revealed by next generation sequencing. Tree Genetics & Genomes.

[ref-9] Folk RA, Mandel JR, Freudenstein JV (2015). A protocol for targeted enrichment of intron-containing sequence markers for recent radiations: a phylogenomic example from *Heuchera* (Saxifragaceae). Applications in Plant Sciences.

[ref-10] Geuten K, Smets E, Schols P, Yuan YM, Janssens S, Küpfer P, Pyck N (2004). Conflicting phylogenies of balsaminoid families and the polytomy in Ericales: combining data in a Bayesian framework. Molecular Phylogenetics and Evolution.

[ref-11] Goremykin VV, Holland B, Hirsch-Ernst KI, Hellwig FH (2005). Analysis of *Acorus calamus* chloroplast genome and its phylogenetic implications. Molecular Biology and Evolution.

[ref-12] Graham SW, Olmstead RG (2000). Utility of 17 chloroplast genes for inferring the phylogeny of the basal angiosperms. American Journal of Botany.

[ref-13] Guisinger MM, Kuehl JNV, Boore JL, Jansen RK (2008). Genome-wide analysis of Geraniaceae plastid DNA reveal unprecedented patterns of increased nucleotide substitutions. Proceedings of the National Academy of Sciences of the United States of America.

[ref-14] Haberle RC, Fourcade HM, Boore JL, Jansen RK (2008). Extensive rearrangements in the chloroplast genome of *Trachelium caeruleum* are associated with repeats and tRNA genes. Journal of Molecular Evolution.

[ref-15] Harris ME, Meyer G, Vandergon T, Vandergon VO (2013). Loss of the acetyl-CoA carboxylase (*accD*) gene in Poales. Plant Molecular Biology Reporter.

[ref-16] Hawkins J, Bodén M (2006). Detecting and sorting targeting peptides with neural networks and support vector machines. Journal of Bioinformatics and Computational Biology.

[ref-17] Hildebrand M, Hallick RB, Passavant CW, Bourque DP (1988). Trans-splicing in chloroplasts: the *rps12* loci of *Nicotiana tabacum*. Proceedings of the National Academy of Sciences of the United States of America.

[ref-18] Hu Q (1994). On the geographical distribution of the Primulaceae. Journal of Tropical and Subtropical Botany.

[ref-19] Jansen RK, Saski C, Lee SB, Hansen AK, Daniell H (2011). Complete plastid genome sequences of three Rosids (*Castanea, Prunus, Theobroma*): evidence for at least two independent transfers of *rpl22* to the nucleus. Molecular Biology and Evolution.

[ref-20] Katoh K, Standley DM (2013). MAFFT multiple sequence alignment software version 7: improvements in performance and usability. Molecular Biology and Evolution.

[ref-63] Kode V, Mudd EA, Iamtham S, Day A (2005). The tobacco plastid *acc*D gene is essential and is required for leaf development. Plant Journal.

[ref-21] Ku C, Hu J-M, Kuo C-H (2013). Complete plastid genome sequence of the basal asterid *Ardisia polysticta* Miq. and comparative analysis of asterid plastid genomes. PLoS ONE.

[ref-22] Lee SS, Jeong WJ, Bae JM, Bang JW, Liu JR, Harn CH (2004). Characterization of the plastid-encoded carboxyltransferase subunit (*accD*) gene of potato. Molecules and Cells.

[ref-23] Li J, Gao L, Chen SS, Tao K, Su YJ, Wang T (2016). Evolution of short inverted repeat in cupressophytes, transfer of *accD* to nucleus in *Sciadopitys verticillata* and phylogenetic position of Sciadopityaceae. Scientific Reports.

[ref-24] Li X, Zhang TC, Qiao Q, Ren ZM, Zhao JY, Yonezawa T, Hasegawa M, Crabbe MJC, Li JQ, Zhong Y (2013). Complete chloroplast genome sequence of holoparasite *Cistanche deserticola* (Orobanchaceae) reveals gene loss and horizontal gene transfer from its host *Haloxylon ammodendron* (Chenopodiaceae). PLoS ONE.

[ref-25] Librado P, Rozas J (2009). DnaSP v5: a software for comprehensive analysis of DNA polymorphism data. Bioinformatics.

[ref-26] Lohse M, Drechsel O, Kahlau S, Bock R (2013). OrganellarGenomeDRAW—a suite of tools for generating physical maps of plastid and mitochondrial genomes and visualizing expression data sets. Nucleic Acids Research.

[ref-27] Ma PF, Zhang YX, Zeng CX, Guo ZH, Li DZ (2014). Chloroplast phylogenomic analyses resolve deep-level relationships of an intractable bamboo tribe Arundinarieae (Poaceae). Systematic Biology.

[ref-28] Magee AM, Aspinall S, Rice DW, Cusack BP, Sémon M, Perry AS, Stefanović S, Milbourne D, Barth S, Palmer JD, Gray JC, Kavanagh TA, Wolfe KH (2010). Localized hypermutation and associated gene losses in legume chloroplast genomes. Genome Research.

[ref-29] Malcomber ST (2002). Phylogeny of *Gaertnera* Lam. (Rubiaceae) based on multiple DNA markers: evidence of a rapid radiation in a widespread, morphologically diverse genus. Evolution.

[ref-30] Martin W, Rujan T, Richly E, Hansen A, Cornelsen S, Lins T, Leister D, Stoebe B, Hasegawa M, Penny D (2002). Evolutionary analysis of *Arabidopsis*, cyanobacterial, and chloroplast genomes reveals plastid phylogeny and thousands of cyanobacterial genes in the nucleus. Proceedings of the National Academy of Sciences of the United States of America.

[ref-31] Martínez-Alberola F, del Campo EM, Lázaro-Gimeno D, Mezquita-Claramonte S, Molins A, Mateu-Andrés I, Pedrola-Monfort J, Casano LM, Barreno E (2013). Balanced gene losses, duplications and intensive rearrangements led to an unusual regularly sized genome in *Arbutus unedo* chloroplasts. PLoS ONE.

[ref-32] Millen RS, Olmstead RG, Adams KL, Palmer JD, Lao NT, Heggie L, Kavanagh TA, Hibberd JM, Gray JC, Morden CW, Calie PJ, Jermiin LS, Wolfe KH (2001). Many parallel losses of *infA* from chloroplast DNA during angiosperm evolution with multiple independent transfers to the nucleus. Plant Cell.

[ref-33] Moore MJ, Dhingra A, Soltis PS, Shaw R, Farmerie WG, Folta KM, Soltis DE (2006). Rapid and accurate pyrosequencing of angiosperm plastid genomes. BMC Plant Biology.

[ref-34] Parks M, Cronn R, Liston A (2009). Increasing phylogenetic resolution at low taxonomic levels using massively parallel sequencing of chloroplast genomes. BMC Biology.

[ref-35] Park S, Jansen RK, Park S (2015). Complete plastome sequence of *Thalictrum coreanum* (Ranunculaceae) and transfer of the *rpl32* gene to the nucleus in the ancestor of the subfamily Thalictroideae. BMC Plant Biology.

[ref-36] Pierleoni A, Martelli PL, Fariselli P, Casadio R (2006). BaCelLo: a balanced subcellular localization predictor. Bioinformatics.

[ref-37] Porebski S, Bailey LG, Baum BR (1997). Modification of a CTAB DNA extraction protocol for plants containing high polysaccharide and polyphenol components. Plant Molecular Biology Reporter.

[ref-38] Raven JA, Allen JF (2003). Genomics and chloroplast evolution: what did cyanobacteria do for plants?. Genome Biology.

[ref-39] Richards J (2003). Primula.

[ref-40] Rousseau-Gueutin M, Huang X, Higginson E, Ayliffe M, Day A, Timmis JN (2013). Potential functional replacement of the plastidic acetyl-CoA carboxylase subunit (*accD*) gene by recent transfers to the nucleus in some angiosperm lineages. Plant Physiology.

[ref-41] Ruhfel BR, Gitzendanner MA, Soltis PS, Soltis DE, Burleigh JG (2014). From algae to angiosperms-inferring the phylogeny of green plants (Viridiplantae) from 360 plastid genomes. BMC Evolutionary Biology.

[ref-42] Shaw J, Lickey EB, Beck JT, Farmer SB, Liu W, Miller J, Siripun KC, Winder CT, Schilling EE, Small RL (2005). The tortoise and the hare II: relative utility of 21 noncoding chloroplast DNA sequences for phylogenetic analysis. American Journal of Botany.

[ref-62] Shaw J, Lickey EB, Schilling EE, Small RL (2007). Comparison of whole chloroplast genome sequences to choose noncoding regions for phylogenetic studies in angiosperms: the tortoise and the hare III. American Journal of Botany.

[ref-43] Shaw J, Shafer HL, Leonard OR, Kovach MJ, Schorr M, Morris AB (2014). Chloroplast DNA sequence utility for the lowest phylogenetic and phylogeographic inferences in angiosperms: the tortoise and the hare IV. American Journal of Botany.

[ref-45] Small I, Peeters N, Legeai F, Lurin C (2004). Predotar: a tool for rapidly screening proteomes for N-terminal targeting sequences. Proteomics.

[ref-46] Son O, Park SJ (2014). Complete chloroplast genome sequence of *Lysimachia coreana* (Primulaceae). Mitochondrial DNA.

[ref-47] Stamatakis A (2006). RAxML-VI-HPC: maximum likelihood-based phylogenetic analysis with thousands of taxa and mixed models. Bioinformatics.

[ref-48] Stevens PF (2012). http://www.mobot.org/MOBOT/research/APweb/.

[ref-49] Timmis JN, Ayliffe MA, Huang CY, Martin W (2004). Endosymbiotic gene transfer: organelle genomes forge eukaryotic chromosomes. Nature Reviews Genetics.

[ref-50] Ueda M, Fujimoto M, Arimura SI, Murata J, Tsutsumi N, Kadowaki K (2007). Loss of the *rp132* gene from the chloroplast genome and subsequent acquisition of a preexisting transit peptide within the nuclear gene in *Populus*. Gene.

[ref-51] Wan S, Mak M-W, Kung S-Y (2014). HybridGO-Loc: mining hybrid features on gene ontology for predicting subcellular localization of multi-location proteins. PLoS ONE.

[ref-52] Wang X, Lu P, Luo Z (2013). GMATo: a novel tool for the identification and analysis of microsatellites in large genomes. Bioinformation.

[ref-53] Wen J, Xiong Z, Nie Z-L, Mao L, Zhu Y, Kan X-Z, Ickert-Bond SM, Gerrath J, Zimmer EA, Fang X-D (2013). Transcriptome sequences resolve deep relationships of the grape family. PLoS ONE.

[ref-54] Wicke S, Schneeweiss GM, dePamphilis CW, Müller KF, Quandt D (2011). The evolution of the plastid chromosome in land plants: gene content, gene order, gene function. Plant Molecular Biology.

[ref-55] Wyman SK, Jansen RK, Boore JL (2004). Automatic annotation of organellar genomes with DOGMA. Bioinformatics.

[ref-56] Wysocki WP, Clark LG, Attigala L, Ruiz-Sanchez E, Duvall MR (2015). Evolution of the bamboos (Bambusoideae; Poaceae): a full plastome phylogenomic analysis. BMC Evolutionary Biology.

[ref-57] Yan HF, Liu YJ, Xie XF, Zhang CY, Hu CM, Hao G, Ge XJ (2015). DNA barcoding evaluation and its taxonomic implications in the species-rich genus *Primula* L. in China. PLoS ONE.

[ref-58] Yang J-B, Li D-Z, Li H-T (2014). Highly effective sequencing whole chloroplast genomes of angiosperms by nine novel universal primer pairs. Molecular Ecology Resources.

[ref-59] Yang Y, Moore MJ, Brockington SF, Soltis DE, Wong GKS, Carpenter EJ, Zhang Y, Chen L, Yan Z, Xie Y, Sage RF, Covshoff S, Hibberd JM, Nelson MN, Smith SA (2015). Dissecting molecular evolution in the highly diverse plant clade Caryophyllales using transcriptome sequencing. Molecular Biology and Evolution.

[ref-60] Yu C-S, Cheng C-W, Su W-C, Chang K-C, Huang S-W, Hwang J-K, Lu C-H (2014). CELLO2GO: a web server for protein subcellular localization prediction with functional gene ontology annotation. PLoS ONE.

[ref-61] Zhang L, Wu W, Yan H-F, Ge X-J (2015). Phylotranscriptomic analysis based on coalescence was less influenced by the evolving rates and the number of genes: a case study in Ericales. Evolutionary Bioinformatics.

