# Supplementary material for: Complete plastid genome sequence of Primula sinensis (Primulaceae): structure comparison, sequence variation and evidence for accD transfer to nucleus
Source: PeerJ. 2016 Jun 28;4:e2101. doi: 10.7717/peerj.2101 (PMC4928469; doi:10.7717/peerj.2101)

**Supplementary materials**

Table S1. Primer sequences designed for verifying the IR-SC boundary and *accD* locus region.

Table S2. Accession numbers of samples downloaded from GenBank involved in phylogenetic analysis.

Table S3. Number of each chloroplast SSR types in two *Primula* species.

Table S4 Detailed information of SSR loci in *Primula sinensis* plastid genome.

Table S5 Detailed information of SSR loci in *Primula poissonii* plastid genome.

Table S6 Detailed results of sequence variation in two *Primula* plastomes.

Figure S1. Reads remapping shows Coverage of *Primula sinensis* chloroplast genome.

Figure S2. This diagram shows the level of divergence between *Primula sinensis* and *Primula poissonii.*

Figure S3. Alignment of pt-*accD* loci of four species from Primulaceae and comparing with the coverage of *Primula sinensis.*

Figure S4. Multiple Alignment of *Primula sinensis* *accD* sequence shows the presence of a deletion to induce the ORF to terminate prematurely.

Table S1. Primer sequences designed for verifying the IR-SC boundary and *accD* locus region.

| Region | primer | sequence | Product length |
| --- | --- | --- | --- |
| *IRa-LSC* | *IRa-LSC_1F* | TTATGAACCCTGTAGACCATCC | 420bp |
|  | *IRa-LSC_1R* | GATGTAGCCAAGTGGATTAAGG |  |
| *IRb-SSC* | *IRb-SSC_1F* | GAATACCGTCACCCATTCTTAC | 1157bp |
|  | *IRb-SSC_1R* | GCATTAATAGCTTGGTCCAC |  |
| *LSC-IRb* | *LSC-IRb_1F* | CATAAGTGTTTCCTCATAGG | 647bp |
|  | *LSC-IRb_1R* | GTAAGCGTCCTGTAGTAAGAG |  |
| *SSC-IRa* | *SSC-IRa_1F* | GAAAGGATCATATATTTGGCGC | 606bp |
|  | *SSC-IRa_1R* | CTAAACAGGAACAAGAGGGATC |  |
| *accD* | *accD_1F* | GGAGTTAGAGTTAGAACACAGG | 1352bp |
|  | *accD_1R* | CTTCTTCGTTTGTTCTTGTC |  |

Table S2. Accession numbers of samples downloaded from GenBank involved in phylogenetic analysis.

| Species | Family | Accession number |
| --- | --- | --- |
| *Primula poissonii* | Primulaceae | NC_024543 |
| *Androsace bulleyana* | Primulaceae | KU513438 |
| *Lysimachia coreana* | Primulaceae | NC_026197 |
| *Ardisia polysticta* | Primulaceae | KC465962 |
| *Actinidia chinensis* | Actinidiaceae | KP297243 |
| *Camellia yunnanensis* | Theaceae | KF156838 |
| *Vaccinium macrocarpon* | Ericaceae | JQ757046 |
| *Arbutus unedo* | Ericaceae | JQ067650 |
| *Agrostemma githago* | Caryophyllaceae | KF527884 |

Table S3. Number of each chloroplast SSR types in two *Primula* species.

|  | Motif | *Primula sinensis* | *Primula poissonii* |
| --- | --- | --- | --- |
| Mononucleotide | T | 75 | 73 |
|  | A | 65 | 52 |
|  | C | 2 | 3 |
|  | G | 6 | 1 |
| Dinucleotide | TA | 16 | 16 |
|  | AT | 8 | 11 |
|  | TC | 7 | 7 |
|  | GA | 5 | 6 |
|  | AG | 5 | 4 |
|  | CT | 3 | 2 |
| Trinucleotide | ATT | 0 | 1 |
|  | TAT | 0 | 1 |
|  | TAA | 1 | 0 |

Table S4 Detailed information of SSR loci in *Primula sinensis* plastid genome.

| SSR type | Numbers of SSR | Distributions |
| --- | --- | --- |
| (A)_8_ | 18 | 4355-4362, 4526-4533, 13492-13499, 27544-27551, 31620-31627, 35852-35859, 41450-41457, 42091-42098, 68041-68048, 68659-68666, 73573-73580, 79746-79753, 80808-80815, 86258-86265, 111685-111692, 112633-112640, 125192-125199, 150700-150707 |
| (A)_9_ | 29 | 4315-4323, 5365-5373, 13594-13602, 15908-15916, 18262-18270, 20024-20032, 28672-28680, 35023-35031, 35525-35533, 46488-46496, 46528-46536, 54099-54107, 56154-56162, 56295-56303, 61630-61638, 64576-64584, 68383-68391, 68400-68408, 73263-73271, 87433-87441, 107500-107508, 107611-107619, 110114-110122, 110713-110721, 111322-111330, 115687-115695, 118263-118271, 132459-132467, 141300-141308 |
| (A)_10_ | 8 | 3663-3672, 23236-23245, 30069-30078, 42199-42208, 45639-45648, 58290-58299, 69306-69315, 75191-75200 |
| (A)_11_ | 3 | 1534-1544, 6708-6718, 46821-46831 |
| (A)_12_ | 4 | 8096-8107, 12426-12437, 46144-46155, 110986-110997 |
| (A)_13_ | 2 | 11562-11574, 13157-13169 |
| (A)_14_ | 1 | 79210-79223 |
| (T)_8_ | 30 | 2121-2128, 5344-5351, 5712-5719, 13669-13676, 14194-14201, 30702-30709, 36381-36388, 41816-41823, 50535-50542, 50671-50678, 53959-53966, 58778-58785, 59449-59456, 61891-61898, 69947-69954, 77169-77176, 80746-80753, 82217-82224, 110537-110544, 112023-112030, 120535-120542, 121400-121407, 121686-121693, 122072-122079, 122578-122585, 122881-122888, 123185-123192, 123440-123447, 124715-124722, 146659-146666 |
| (T)_9_ | 18 | 4922-4930, 7382-7390, 7705-7713, 8620-8628, 9911-9919, 18014-18022, 31382-31390, 47331-47339, 80629-80637, 91616-91624, 100457-100465, 110425-110433, 117664-117672, 120228-120236, 123227-123235, 124955-124963, 125416-125424, 145483-145491 |
| (T)_10_ | 8 | 6252-6261, 25830-25839, 53519-53528, 66562-66571, 68825-68834, 76313-76322, 123504-123513, 124730-124739 |
| (T)_11_ | 11 | 18120-18130, 31047-31057, 43322-43332, 67559-67569, 67619-67629, 73682-73692, 78108-78118, 121911-121921, 123156-123166, 124044-124054, 125162-125172 |
| (T)_12_ | 4 | 3625-3636, 56206-56217, 72134-72145, 78626-78637 |
| (T)_13_ | 2 | 9779-9791, 117560-117572 |
| (T)_15_ | 2 | 8443-8457, 50466-50480 |
| (G)_8_ | 3 | 46497-46504, 46537-46544, 69869-69876 |
| (G)_9_ | 1 | 45649-45657 |
| (G)_10_ | 1 | 101485-101494 |
| (G)_13_ | 1 | 35506-35518 |
| (C)_9_ | 1 | 120442-120450 |
| (C)_10_ | 1 | 131430-131439 |
| (AT)_4_ | 5 | 24053-24060, 35594-35601, 44586-44593, 54123-54130, 60676-60683 |
| (AT)_5_ | 2 | 19505-19514, 110473-110482 |
| (AT)_7_ | 1 | 79780-79793 |
| (AG)_4_ | 5 | 30160-30167, 74488-74495, 92768-92775, 99922-99929, 129304-129311 |
| (TA)_4_ | 14 | 2160-2167, 6678-6685, 12296-12303, 12943-12950, 41306-41313, 46358-46365, 46367-46374, 57903-57910, 76017-76024, 76232-76239, 90819-90826, 94943-94950, 137974-137981, 142098-142105 |
| (TA)_6_ | 1 | 7029-7040 |
| (TA)_7_ | 1 | 64594-64607 |
| (TC)_4_ | 7 | 48597-48604, 69225-69232, 121370-121377, 132994-133001, 147471-147478, 148458-148465, 148470-148477 |
| (CT)_4_ | 3 | 18963-18970, 103613-103620, 140149-140156 |
| (GA)_4_ | 5 | 34762-34769, 84447-84454, 84459-84466, 85446-85453, 114653-114660 |
| (TAA)_4_ | 1 | 44144-44155 |

Table S5 Detailed information of SSR loci in *Primula poissonii* plastid genome.

| SSR type | Numbers of SSR | Distributions |
| --- | --- | --- |
| (A)8 | 30 | 139-146, 1480-1487, 2698-2705, 3585-3592, 7652-7659, 12943-12950, 13238-13245, 17993-18000, 21681-21688, 27174-27181, 30002-30009, 35846-35853, 36106-36113, 41717-41724, 46450-46457, 54083-54090, 54588-54595, 56645-56652, 65032-65039, 69829-69836, 71372-71379, 75066-75073, 87643-87650, 108634-108641, 112374-112381, 112736-112743, 113938-113945, 126215-126222, 142012-142019, 151580-151587 |
| (A)9 | 13 | 5291-5299, 11315-11323, 28326-28334, 45866-45874, 47106-47114, 54127-54135, 82112-82120, 87756-87764, 88932-88940, 110162-110170, 111779-111787, 112136-112144, 116766-116774 |
| (A)10 | 6 | 7824-7833, 62535-62544, 68721-68730, 74749-74758, 112022-112031, 133409-133418 |
| (A)11 | 1 | 81291-81301 |
| (A)12 | 1 | 6581-6592 |
| (A)13 | 1 | 80712-80724 |
| (T)8 | 32 | 2063-2070, 3847-3854, 5488-5495, 8493-8500, 13610-13617, 13943-13950, 27122-27129, 36678-36685, 41501-41508, 42085-42092, 50530-50537, 54451-54458, 58419-58426, 59776-59783, 70865-70872, 81928-81935, 83522-83529, 93090-93097, 111598-111605, 113083-113090, 113577-113584, 113643-113650, 115284-115291, 118628-118635, 118881-118888, 122835-122842, 123721-123728, 124015-124022, 124307-124314, 125735-125742, 126468-126475, 147459-147466 |
| (T)9 | 23 | 4883-4891, 6167-6175, 7272-7280, 17744-17752, 28924-28932, 30566-30574, 41367-41375, 50923-50931, 56709-56717, 78711-78719, 80142-80150, 82055-82063, 118707-118715, 121336-121344, 123221-123229, 124349-124357, 124550-124558, 125071-125079, 125560-125568, 125750-125758, 125978-125986, 146169-146177, 147345-147353 |
| (T)10 | 11 | 15953-15962, 26765-26774, 43580-43589, 54012-54021, 58488-58497, 59188-59197, 69069-69078, 77857-77866, 79613-79622, 101691-101700, 124529-124538 |
| (T)11 | 4 | 17850-17860, 42575-42585, 123060-123070, 126185-126195 |
| (T)12 | 2 | 25549-25560, 34957-34968 |
| (T)13 | 1 | 48362-48374 |
| (C)8 | 2 | 36098-36105, 71952-71959 |
| (C)9 | 1 | 108720-108728 |
| (G)9 | 1 | 76593-76601 |
| (AG)4 | 4 | 30746-30753, 94175-94182, 101156-101163, 130255-130262 |
| (AT)4 | 8 | 6472-6479, 19148-19155, 23772-23779, 35912-35919, 44817-44824, 54611-54618, 61580-61587, 111535-111542 |
| (AT)5 | 2 | 29605-29614, 46509-46518 |
| (AT)7 | 1 | 123286-123299 |
| (TA)4 | 13 | 2102-2109, 6912-6919, 12058-12065, 58827-58834, 67474-67481, 77562-77569, 77776-77783, 92303-92310, 96329-96336, 119278-119285, 124119-124126, 138773-138780, 142799-142806 |
| (TA)5 | 1 | 47071-47080 |
| (TA)6 | 1 | 46626-46637 |
| (TA)7 | 1 | 45747-45760 |
| (TC)4 | 7 | 48962-48969, 70651-70658, 129935-129942, 133945-133952, 148352-148359, 149339-149346, 149351-149358 |
| (GA)4 | 6 | 35093-35100, 85751-85758, 85763-85770, 86750-86757, 105167-105174, 115695-115702 |
| (CT)4 | 2 | 104847-104854, 140927-140934 |
| (TAT)4 | 1 | 76796-76807 |
| (ATT)4 | 1 | 80164-80175 |

Table S6 Detailed results of sequence variation in two *Primula* plastomes.

| No. | Region | Aligned position | Aligned length | NSs | Indels | PIC | Pi |
| --- | --- | --- | --- | --- | --- | --- | --- |
| 1 | *trnH-psbA* | 76-289 | 213 | 39 | 3 | 42 | 0.24375 |
| 2 | *psbA-matK* | 1351-1878 | 527 | 36 | 4 | 40 | 0.071429 |
| 3 | *matK* | 1879-3416 | 1539 | 88 | 1 | 89 | 0.057743 |
| 4 | *trnK-rps16* | 4175-5016 | 841 | 71 | 11 | 82 | 0.095816 |
| 5 | *rps16-trnQ* | 6139-6963 | 825 | 216 | 18 | 234 | 0.305516 |
| 6 | *psbK-psbI* | 7573-7929 | 356 | 26 | 5 | 31 | 0.078078 |
| 7 | *trnS-trnG* | 8243-8951 | 708 | 70 | 8 | 78 | 0.110063 |
| 8 | *trnG intron* | 8974-9705 | 731 | 39 | 2 | 41 | 0.056034 |
| 9 | *atpH-atpI* | 13739-14486 | 747 | 57 | 6 | 63 | 0.078082 |
| 10 | *rps2-rpoC2* | 16161-16395 | 234 | 17 | 2 | 19 | 0.082927 |
| 11 | *rpoB-trnC* | 26786-27978 | 1192 | 74 | 12 | 86 | 0.074522 |
| 12 | *trnC-petN* | 28059-28518 | 459 | 44 | 3 | 47 | 0.101852 |
| 13 | *petN-psbM* | 28608-29617 | 1009 | 66 | 13 | 79 | 0.078853 |
| 14 | *psbM-trnD* | 29743-30984 | 1241 | 64 | 12 | 76 | 0.078431 |
| 15 | *trnT-psbD* | 32116-33414 | 1298 | 67 | 13 | 80 | 0.06381 |
| 16 | *psbZ-rps14* | 36721-37429 | 708 | 48 | 9 | 57 | 0.07218 |
| 17 | *psaA-pafI* | 42345-42941 | 596 | 40 | 10 | 50 | 0.074212 |
| 18 | *pafI-trnS* | 44900-45777 | 877 | 63 | 9 | 72 | 0.07563 |
| 19 | *trnT-trnL* | 47203-48073 | 870 | 62 | 8 | 70 | 0.097946 |
| 20 | *trnL-trnF* | 48110-49072 | 962 | 55 | 12 | 67 | 0.066345 |
| 21 | *trnF-ndhJ* | 49145-49823 | 678 | 45 | 9 | 54 | 0.078125 |
| 22 | *ndhC-trnV* | 51503-52454 | 952 | 146 | 18 | 164 | 0.192612 |
| 23 | *pafII-cemA* | 61660-62187 | 527 | 48 | 9 | 57 | 0.108352 |
| 24 | *petA-psbJ* | 64077-65108 | 1031 | 70 | 13 | 83 | 0.116086 |
| 25 | *psbE-petL* | 65889-67008 | 1119 | 81 | 8 | 89 | 0.084025 |
| 26 | *petL-trnP* | 67104-67770 | 666 | 41 | 8 | 49 | 0.064669 |
| 27 | *trnP-psaJ* | 67844-68251 | 406 | 28 | 6 | 34 | 0.075676 |
| 28 | *psaJ-rpl33* | 68386-68888 | 502 | 32 | 5 | 37 | 0.06823 |
| 29 | *rpl20-clpP* | 70196-71229 | 1033 | 61 | 6 | 67 | 0.05957 |
| 30 | *clpP-psbB* | 72404-73836 | 1434 | 69 | 11 | 80 | 0.053118 |
| 31 | *rpl36-rps8* | 81186-81542 | 356 | 102 | 6 | 108 | 0.331169 |
| 32 | *ndhF_partial* | 111520-111963 | 445 | 29 | 1 | 30 | 0.066514 |
| 33 | *ndhF-rpl32* | 113483-114384 | 901 | 78 | 15 | 93 | 0.09319 |
| 34 | *rpl32-trnL* | 114546-114981 | 435 | 59 | 7 | 66 | 0.154047 |
| 35 | *ccsA-ndhD* | 116152-116362 | 210 | 33 | 5 | 38 | 0.168367 |
| 36 | *psaC-ndhG* | 118266-119088 | 823 | 52 | 4 | 56 | 0.066667 |
| 37 | *ndhG-ndhI* | 119619-119969 | 350 | 27 | 4 | 31 | 0.083077 |
| 38 | *ndhA_intron* | 121117-122273 | 1156 | 66 | 13 | 79 | 0.066398 |
| 39 | *rps15-ycf1* | 124360-124746 | 387 | 38 | 5 | 43 | 0.111765 |
| 40 | *ycf1_partial1* | 125548-126529 | 982 | 68 | 3 | 71 | 0.072805 |
| 41 | *ycf1_partial2* | 126671-128233 | 1562 | 107 | 4 | 111 | 0.075299 |
| 42 | *rbcL+matK+ITS* | n.a. | 3594 | 225 | 7 | 232 | 0.06299 |

Figure S1. Reads remapping shows Coverage of *Primula sinensis* chloroplast genome. Vertical axis represents the common logarithm (log10) of each position coverage value. Horizontal axis represents each position of corrected complete chloroplast genome.


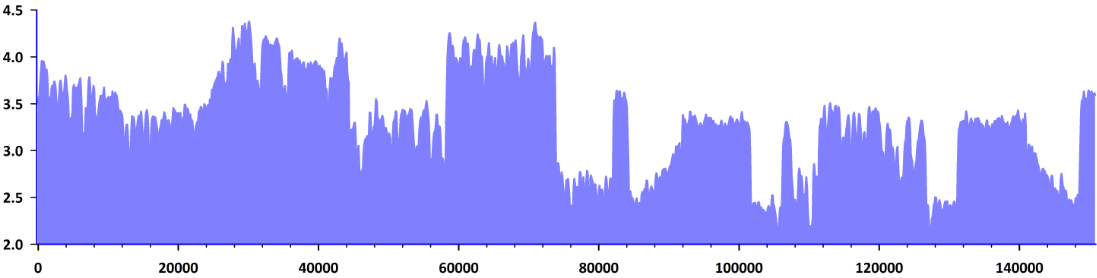


Figure S2. This diagram shows the level of divergence between *Primula sinensis* and *Primula poissonii.* The horizontal axis represents coordinate position of each sliding window within the plastome alignment of two *Primula* species. The vertical scale indicates the Pi value of each window region for illustrating divergence levels. Red dashed line shows the criterion of divergence value corresponding to the concatenated sequence of three barcode regions (*rbcL*+*matK*+*ITS*). Above peak graph, black arrows represent the annotations of coding genes indicating the position and direction of each gene in this alignment.


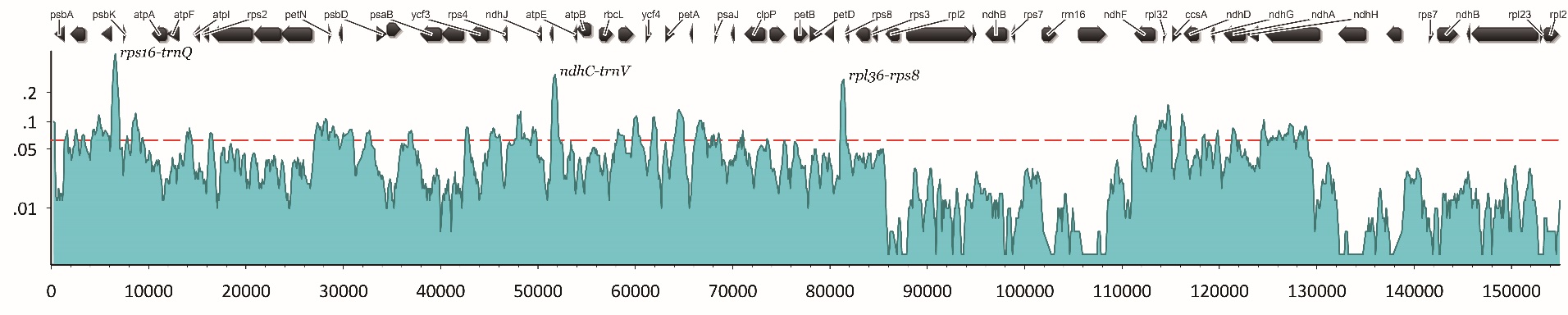


Figure S3. Alignment of pt-*accD* loci of four species from Primulaceae and comparing with the coverage of *Primula sinensis.*


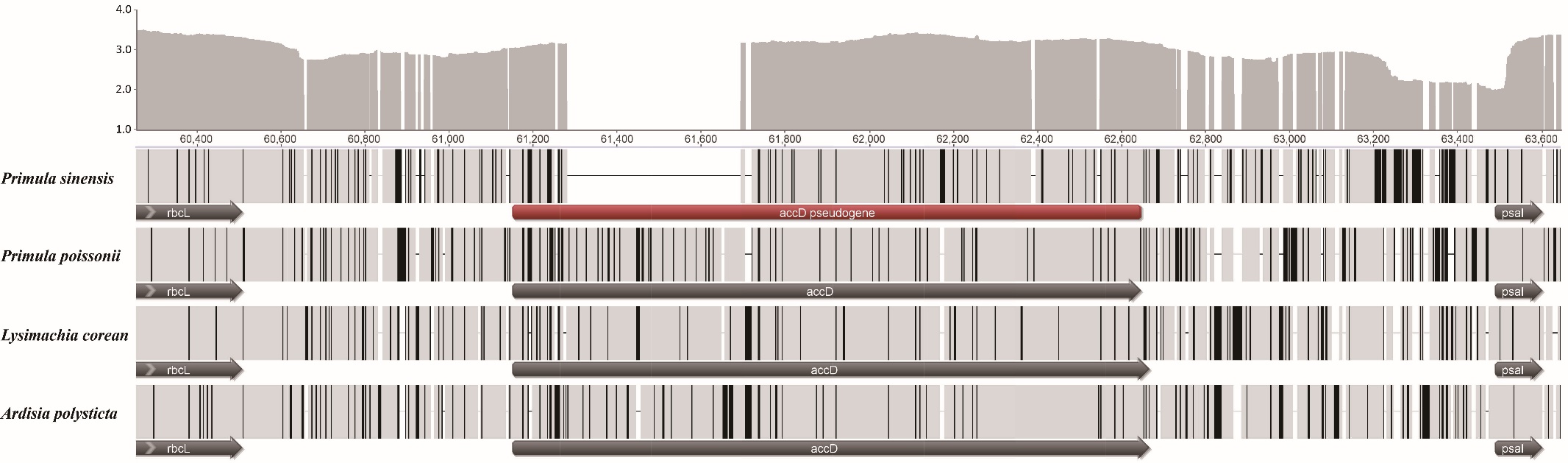


Figure S4. Multiple alignment of *Primula sinensis* *accD* sequence shows the presence of a deletion to induce the ORF to terminate prematurely. Red color indicates the reduced ORF without C-terminal functional structure sequence.


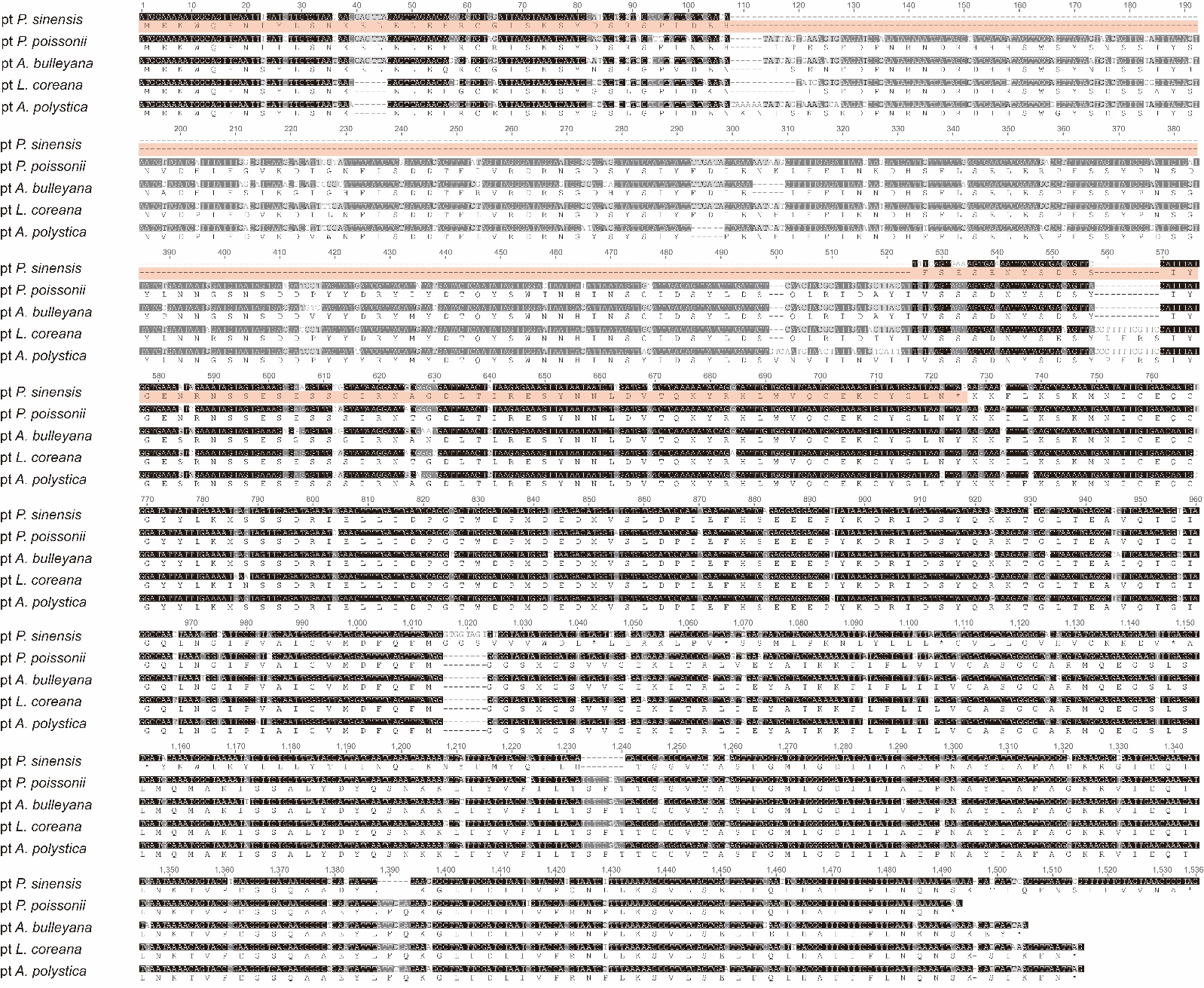

Supplement: Supplemental Information 1 [file peerj-04-2101-s001.docx]
